# Developing embodied cognition: insights from children’s concepts and language processing

**DOI:** 10.3389/fpsyg.2014.00506

**Published:** 2014-05-28

**Authors:** Michele Wellsby, Penny M. Pexman

**Affiliations:** Language Processing Laboratory, Department of Psychology, University of Calgary, CalgaryAB, Canada

**Keywords:** developmental science, embodied cognition, language development, sensorimotor processing, action, concepts

## Abstract

Over the past decade, theories of embodied cognition have become increasingly influential with research demonstrating that sensorimotor experiences are involved in cognitive processing; however, this embodied research has primarily focused on adult cognition. The notion that sensorimotor experience is important for acquiring conceptual knowledge is not a novel concept for developmental researchers, and yet theories of embodied cognition often do not fully integrate developmental findings. We propose that in order for an embodied cognition perspective to be refined and advanced as a lifelong theory of cognition, it is important to consider what can be learned from research with children. In this paper, we focus on development of concepts and language processing, and examine the importance of children's embodied experiences for these aspects of cognition in particular. Following this review, we outline what we see as important developmental issues that need to be addressed in order to determine the extent to which language and conceptual knowledge are embodied and to refine theories of embodied cognition.

Embodied cognition (EC) is a broad term used to describe a class of theories within cognitive science, many of which emphasize the importance of sensorimotor experience gained through our bodily interactions with the environment for acquiring and representing conceptual knowledge (Borghi and Cimatti, 2010). That is, contrary to classical cognitive theories, which deemphasized the importance of the body for cognitive processing and posited that cognition strictly involved the processing of abstract and amodal symbols, EC theories tend to assume that our actions and bodily experiences are crucial to our cognitive processing. According to EC theories, direct sensorimotor interactions are essential for gaining knowledge and developing cognitive capabilities (Engel et al., 2013), and higher order and offline cognitive processing (i.e., removed from the environment) involve re-enactment of the bodily states from previous experience (Foglia and Wilson, 2013).

Theories of EC have become a prominent way of conceptualizing cognitive processing and have been particularly influential in reconceptualising and explaining adult language processing. A large number of studies have now provided evidence that when comprehending language, adults simulate the meaning implied in words and sentences [e.g., implied motion (Glenberg and Kaschak, 2002), object orientation (Stanfield and Zwaan, 2001), object affordances (Myung et al., 2006)]. Thus, adults use sensorimotor information gained through their experiences with the world to represent concepts and comprehend language. There continues to be debate, however, about whether sensorimotor experiences comprise conceptual knowledge and language or whether accessing this information merely activates sensorimotor areas epiphenomenally. In the adult literature, there are now a number of variants of EC theories that posit different degrees of embodiment and disembodiment (e.g., Mahon and Caramazza, 2008; Meteyard et al., 2012). These theories can be viewed along a continuum ranging from strongly embodied to disembodied, differing in their assumptions about the nature of the relationship between sensorimotor and cognitive processing.

The disembodied end of the spectrum is represented by what is essentially the classical cognitive perspective described above, which posits that sensorimotor experiences are not involved in cognitive processing (Meteyard et al., 2012). From a developmental perspective, this end of the spectrum would be represented by the view that, while sensorimotor experiences might be important for infants’ earliest learning, cognition becomes progressively more abstract and less embodied with development. At the other end of the spectrum, a strong embodied account suggests that cognition is constituted in action and sensorimotor processing (Glenberg and Gallese, 2012), and that our conceptual representations are dependent on sensorimotor experiences. From a strong embodied perspective, cognitive processing involves a recreation of direct sensory experience (Meteyard et al., 2012), in childhood and beyond.

An alternative view is taken by secondary embodiment theories, which propose that sensorimotor areas of the brain are activated as a by-product of cognitive processing, through spreading activation (Mahon and Caramazza, 2008). From this perspective, sensorimotor activation during cognitive processing is a passive consequence of, as opposed to a necessity for, representing a concept. Finally, a weak embodied account suggests that conceptual representations are partially comprised of sensorimotor knowledge, as sensorimotor interactions help to ground concepts during initial knowledge acquisition. However, activation of this same sensorimotor information is not required for conceptual processing; rather, representations are abstracted from the initial experience, and then are organized to form conceptual knowledge (Gennari, 2012; Meteyard et al., 2012).

There is also growing support for hybrid or pluralist theories that add to or combine different components of the embodiment spectrum (e.g., Paivio, 1990; Barsalou et al., 2008; Louwerse and Jeuniaux, 2010). For example, Dove (2011) proposed that an embodied approach, in which conceptual representations consist mainly of simulation of previous sensorimotor experience (perceptual symbols), is more useful for certain concepts than others; specifically, for concrete concepts as compared to abstract concepts. Dove emphasized that in order for an embodied theory to adequately provide an explanation of abstract concepts, language and linguistic symbols would be important. Thus, concepts comprise both sensorimotor representations, gained through previous embodied experience, and also what Dove called dis-embodied representations, gained from our experience with language. From this perspective, our knowledge of concepts is not only comprised of our sensorimotor experience but also how we use language. By this view, concrete concepts are comprised of embodied sensorimotor information from previous interactive experience with objects and the environment (perceptual symbols), as well as dis-embodied sensorimotor information from our experience using language (linguistic symbols), whereas our understanding of abstract concepts is mainly comprised of information from our experience with language.

Taking a slightly different perspective, Pulvermuller and Garagnani (2014) proposed that different types of cognitive processing could involve different degrees of embodiment, such that while long-term memory is embodied and is grounded in sensorimotor systems, working memory relies less on those systems. In a similar vein, Zwaan (2014) proposed that rather than arguing for or against a particular version of embodiment, we instead need to investigate the relative importance of sensorimotor information and symbolic representations in different contexts for language processing. In particular, language comprehension that is relatively more embedded in the environment will likely involve more embodied processing.

Thus, it is evident that there are multiple theories of embodiment, which differ in how much emphasis is placed on sensorimotor experiences for conceptual and language processing. It seems possible that a lifespan perspective could afford new insights on these issues by examining the developmental trajectory of how sensorimotor experiences shape language and conceptual processing. In addition, rather than simply taking an “embodied versus disembodied” stance, it is essential to determine specific details surrounding when and how sensorimotor representations are involved in language and conceptual processing (Willems and Francken, 2012). As we will discuss below, children initially use sensorimotor information to gain conceptual knowledge. By examining how and when sensorimotor information is important for children’s linguistic and conceptual understanding, and determining if and when they shift away from a reliance on this sensorimotor knowledge as their cognition becomes more sophisticated and more abstract, it seems likely that developmental research could help advance EC theories more generally.

As described above, EC theories can be viewed along a continuum with regards to the emphasis placed on the role of embodied experience, and numerous studies have demonstrated that embodied knowledge plays some sort of role in adult concepts and language processing. However, there has been less research conducted to examine embodied effects in children’s conceptual and linguistic processing, and less discussion of the implications for EC theories in research examining cognitive development. Although developmental research does not often use the term “embodied cognition” when describing children’s cognitive processing, the notion that sensorimotor experience is essential to conceptual and linguistic knowledge is not a novel idea in the developmental field. Kontra et al. (2012) proposed that “theories of embodied cognition have the potential to deepen our understanding of the mechanisms underlying early developmental changes driven by action experience” (p. 738); in addition, we propose that to refine theories of EC, it is essential to consider the insights that can be gleaned from developmental research, examining children’s sensorimotor experiences and how those experiences shape their knowledge.

In this paper, we will first review developmental theories and recent evidence from the developmental literature that highlight the importance of sensorimotor experience early on in childhood for the development of later cognitive skills and abilities. By sensorimotor experience, we refer to a range of experiences that typically involve an action being performed on an object, either by a child directly, or through observation of another’s action. The experience is multisensory, primarily derived from visual, tactile, and proprioceptive senses. We have chosen to focus on this characterization of sensorimotor experience (which is quite broad) because this is what has typically been examined in child development research. Certainly, grounding of conceptual information could involve other systems, such as emotions (e.g., Pulvermuller, 2013), but there is as yet little research on how children ground the meaning of language and concepts through emotion (we return to this in our final “issues to be addressed” section). Additionally, although there are numerous aspects of development we could examine, we limited our review to emphasize research on children’s language and conceptual processing. These areas will be our focus because language and concepts have been at the center of much of the debate between strong, weak, and secondary theories of EC (Zwaan, 2014). We first review these findings, and then describe what we see as pertinent issues that need to be addressed in order that EC theories can be further refined and advanced as theories of lifelong cognitive development.

## THE IMPORTANCE OF SENSORIMOTOR EXPERIENCE IN DEVELOPMENT

Although EC theories have not been prominent in the developmental literature, the notion that sensorimotor experience is essential to child development is certainly not a new concept. The proposal that sensorimotor information initially drives cognitive development was an important aspect of Piaget’s work, and he emphasized the influential role of children’s interactions with their environment (Piaget, 1952; Laakso, 2011). Piaget argued that in early infancy, sensorimotor experiences are an essential aspect of learning, and later cognitive processes develop from these sensorimotor abilities. The general idea emphasized in the developmental literature is that infants are embodied learners, and use sensorimotor information to gain knowledge about their world (Laakso, 2011). It has been proposed that infants develop a representational system as a result of early perceptual and motor interactions with their environment (Meltzoff, 1990). These early representations are considered the building blocks that allow embodied learning to continue throughout childhood. Whereas Piaget proposed that children go on to develop concepts that are independent of their sensorimotor experience, others have argued that as children develop increased cognitive and physical capabilities, their sensorimotor interactions with the environment continue to be important for language processing and increased conceptual understanding (Gibbs, 2006).

While few would challenge the claim that infants and young children initially use sensorimotor knowledge and interactions with their environment to acquire information, the extent to which embodied experience is relevant for higher-order cognitive functioning (e.g., language processing) in childhood has been less widely considered. Given the results of adult studies it seems likely, however, that EC theories can ultimately explain how sensorimotor knowledge is beneficial for early sensory learning in infancy, for motor and action development through childhood, and for language and higher-order cognitive functioning in school-aged children (Kontra et al., 2012).

Further, a theory emphasizing the importance of embodiment across the life-span would propose that the role of embodiment in conceptual processing is always present; the influence of sensorimotor experiences does not stop or change fundamentally throughout development, it just may become more refined and flexible over time (Antonucci and Alt, 2011). Indeed, the role of embodiment in conceptual processing is considered by some developmental theorists to be continuous, as conceptual representations across the lifespan are composed of perceptual and action experiences (Thelen, 2008), and the successive development of sensation, action, and language across childhood into adulthood is influenced by the experiences that a child has in their environment (Borghi and Cimatti, 2010). Embodied experiences contribute to a dynamic grounding of cognition over the lifespan that allows children and adults to learn language and represent concepts based on previous sensorimotor interactions (Thelen, 2008). Children interact with their environment and learn concepts, and language can then be mapped onto these representations (e.g., Glenberg and Gallese, 2012). There is evidence that children’s sensorimotor experience and actions towards objects directly influence their word and concept learning (e.g., O’Neill et al., 2002; Smith, 2005). Although it appears likely that conceptual knowledge is grounded in the environment from infancy onward (Zwaan and Kaschak, 2009), with sensorimotor interactions continuously shaping cognitive processing, there has been little integration of developmental findings with theories of EC to explain the relationship between cognition and bodily experience across development (Gabbard, 2013).

There is longitudinal evidence for the relationship between children’s early sensorimotor (in particular, action) experiences and later higher-order cognitive functioning. For example, Bornstein et al. (2013) recently reported the results of a longitudinal study conducted to examine motor exploration behavior in infancy and how this behavior predicted academic abilities in adolescence. Bornstein et al. (2013) measured the motor-exploratory competence (movement, balance, and locomotion) and exploratory activity of five-month-old infants. Longitudinal data showed that infants with higher scores on the motor exploration variables at 5-months of age had higher scores on intellectual and academic measures at 4-, 10-, and 14-years of age. While there are likely multiple mediating factors, it is probable that the infants with relatively high motor competency and exploratory behavior had more opportunities for sensorimotor interactions with objects and with their environment. For instance, infants who are able to sit and maintain balance while manipulating objects are able to acquire multimodal sensorimotor information about objects (Smith, 2013). This increased embodied experience could facilitate sustained attention, richer interactive experiences, and more instances of adults labeling objects, which all contribute to greater knowledge of objects in the environment. In turn, vocabulary, attention, and knowledge could all be enhanced, resulting in positive long-term cognitive outcomes like those observed by Bornstein et al. (2013). Thus, evidence suggests that there are benefits of exploration and increased motor activity in infancy (i.e., embodied interactions) for later cognitive development. Of course, this type of research does not allow us to make inferences about the types of embodied experiences that are most important, but that is better achieved by the experimental studies on this topic, reviewed next.

## THE INFLUENCE OF EARLY SENSORIMOTOR EXPERIENCE ON CHILDREN’S CONCEPTS AND WORD LEARNING

### NOUNS

It is widely agreed that before children acquire language, they build conceptual representations based on their sensorimotor experiences with the world (Antonucci and Alt, 2011). Once infants are able to sit and manipulate objects, they are able to acquire information about objects based on motor, tactile, visual, and auditory input (Smith, 2013). Through active exploration with the environment, children develop an increased understanding of the functions of objects and how they can be manipulated. This knowledge of semantic features and object affordances helps children to differentiate objects more easily, and eventually to learn words by mapping labels onto representations based on previous experiences (Scofield et al., 2009).

Findings from research examining infants’ and children’s interactions demonstrate effects of specific types of sensorimotor experience on categorization and word learning. In particular, the manner in which children act on objects, with regards to actions performed and sensorimotor experience obtained, influences how these objects are conceptualized (Smith, 2005). In Smith’s (2005) study, 2-year-old children were introduced to an exemplar object called a “wug,” which the experimenter labeled while moving the object either horizontally or vertically. Some of the children were also given the opportunity to move the object themselves, in the same direction. Following this, children were asked to select the wug from two novel objects: an object that was the same height as the exemplar, but extended horizontally, and an object that was the same diameter as the exemplar, but extended vertically. For the children who had manipulated the object themselves, there was an interaction between the direction they had moved the object and the object they selected as the wug: children who had watched and then moved the wug horizontally chose as the wug the novel object that was extended horizontally, and vice versa. Interestingly, there was no such effect for the children who only watched the experimenter move the objects.

Smith (2005) offered an embodied explanation for these findings, by proposing that the way the children manipulated and acted on the object comprised part of their conceptual representation for that object. For the children who interacted with the object, the sensorimotor experience created a mental representation of the object based on the action performed, which influenced their judgment of the objects’ shape; this motor information was later simulated when the children viewed the novel objects and had to make a categorization decision.

Other studies have also demonstrated that the way in which objects are held and manipulated influences the aspects of that object that are relevant for children’s categorization (Smith et al., 2007). In one study, 2-year-old children were taught a novel label for an object with a hinge and were given the opportunity to interact with the object. When children were then presented with similarly shaped objects without a hinge and objects that differed in shape but had a hinge, the children were more likely to extend the novel object label to the other objects with hinges. Thus, the functional knowledge gained through interaction with objects can determine how objects and categories are formed.

Additional research has examined how spatial location and body positioning influence word learning (Smith and Samuelson, 2010). Children between 18 and 24 months of age were presented with two unlabeled objects one at a time, one to their right, and one to their left. Following this, the objects were removed and a label was provided to one of the empty locations where an object had previously been presented (e.g., “modi”). When the children were later shown both of the objects in new locations and asked to select the named object (“where is the modi?”), the majority of the children selected the object that had been presented in the location where the label was provided. Thus, children associated the object’s location with its label, suggesting that visuospatial experience with the object’s location (and not just with the object itself) influences word learning. Interestingly, changing the children’s posture from sitting to standing decreased their ability to map the label to the object. This finding suggested, further, that children’s body posture also played a role in linking label to object.

To further examine the influence of sensory experience and body posture on object learning, Morse et al. (2010) extended the Smith and Samuelson (2010) paradigm to the field of developmental robotics. Morse et al. (2010) replicated the Smith and Samuelson (2010) experiments using a robot, and reported that the robot’s categorization performance was comparable to the children’s performance in the Smith and Samuelson study. Taken together, these results indicate that sensory representations, as well as proprioceptive information about body posture, are both important factors when learning to categorize and map labels to objects. The robotics simulations provide additional insight about the sensory representations that are involved in category learning.

### VERBS

One general theme in the developmental literature is that interactions with the environment play an important role in verb learning. For instance, according to Glenberg and Gallese (2012), children’s understanding of verbs is grounded in bodily actions and sensorimotor experiences. That is, a verb like “give” would be understood in infancy from concrete experiences of giving objects to parents/caregivers; the meaning would be grounded in these actions. Children’s bodily actions towards other people are also related to their understanding of abstract verbs, such as “love” or “hate,” that do not appear to being grounded in one specific action. These verbs can be associated with observable bodily behaviors (such as showing affection) that can help ground understanding of the emotional content associated with the verb meaning (Smith et al., 2007).

Moving beyond infancy, the role of sensorimotor experience in verb learning has been directly examined in young children, with findings indicating that there are differences in brain activation as a result of whether verbs were learned through self-performed or observed actions (James and Bose, 2011; James and Swain, 2011). Children aged 5- to 7-years were taught novel verbs either by actively performing the action while repeating the verb label out loud, or by watching an experimenter perform the action while the experimenter repeated the verb label. Then, children were presented with auditory and visual information from the objects (e.g., verb label, video of the action being performed) during fMRI scanning. When the action label was auditorily presented, motor areas in the brain (including regions associated with grasping objects) were activated only for the verbs the children had learned through self-action, not for verbs learned through passive observation (James and Swain, 2011). The same pattern of findings was observed when viewing videos of the actions, with greater activation for actively learned verbs in areas associated with tool use, integrating motor information, and visual processing (James and Bose, 2011). These findings suggest that sensorimotor movements evoked when learning language are reactivated during recognition. Further, it appears that in order for perception and action to become linked and for motor representations to be re-activated when action verbs are heard, children may need to have actively interacted with objects.

### ADJECTIVES

Studies have also examined the influence of children’s sensorimotor experience with objects when learning other parts of speech, such as adjectives. For example, two-year-old children were taught novel adjectives (e.g., spiny, spongy) by an adult using either referential gestures toward an object (i.e., pointing to an object) or descriptive gestures (e.g., using tactile gestures, such as squeezing the spongy object; O’Neill et al., 2002). On each trial, an animal toy was given to the child and an adjective was provided. When providing the adjective, the experimenter either gestured with the toy to illustrate the property or pointed to the toy. Thus, the descriptive gestures provided sensorimotor information about the adjective, through observation as well as any actions the child made toward the toy. In contrast, the referential point did not provide this sensorimotor information and only acted as an attentional cue.

On test trials, children were presented with two toys and asked for one displaying a specific property (e.g., “Give me the lumpy toy”). The children who were taught adjectives using descriptive gestures performed better at test, and additionally, descriptive gestures were especially helpful at teaching adjectives that did not correspond to visual properties. That is, observation of descriptive gestures was more beneficial for teaching adjectives such as lumpy and spongy, where tactile experiences are essential to meaning, as compared to adjectives such as spiny, for which the meaning can be inferred through visual inspection. Interestingly, more accurate performance in the test trials was not related to the amount of sensorimotor interaction the children had during the teaching trials. There was, however, a positive relationship between performance and interaction at test. That is, the children who were taught adjectives by viewing descriptive gestures used this sensory information in the test trials to perform the gesture themselves and, presumably, to determine which object fit with the adjective they were asked to identify (O’Neill et al., 2002). Thus, although the children in both conditions interacted with the objects during the training trials, the children who were in the descriptive gesture condition seemed to use these gestures as a cue to focus on that specific property of the object. It seems likely that the children who observed descriptive gestures gained tactile information about the objects that allowed them to ground the meaning of the adjective.

### QUALIFYING THE BENEFITS OF SENSORIMOTOR EXPERIENCE

Although there is evidence that sensorimotor experience supports children’s word learning, there is also evidence that this is not always the case. Tare et al. (2010) examined how manipulative features influenced children’s learning of novel animal names from picture books. That is, 20-month-old children were taught labels for novel animals using one of three picture books: a book with drawings of animals, a book with photos of animals, or a book with drawings of animals and manipulative features with which the children could interact (e.g., a flap to pull up to reveal an animal). The children who were taught the animal name using a picture book with realistic photos demonstrated the most accurate learning, while children who were read the picture book with manipulative features had the least accurate learning. A similar pattern was observed in a second study with 30- and 36-month-olds who were read the same books but were also taught facts about the animals (e.g., birds like to eat worms). These findings indicate that having children interact with attention-capturing features like pop up flaps may not always be beneficial for word learning, particularly when the sensorimotor experience obtained does not correspond with the information to be learned.

It is also possible that certain kinds of sensory information may be more relevant for learning certain word classes. For instance, it has been suggested that functional information may be relatively more important than sensory information for distinguishing between inanimate objects (Warrington and Shallice, 1984). Results from recent robotics work suggest additional differences between the information that is important for learning words of different classes. In a study by Yuruten et al. (2013), a robot interacted with objects using different manipulations to learn nouns and adjectives. The authors examined the relevance of different object features, and determined that object affordances were more important for learning adjectives, while object appearance was more important for learning nouns. This suggests, again, that specific kinds of sensorimotor experiences are useful for learning different kinds of concepts.

While an EC account would propose that previous interactions with an object comprise the representation for that object (e.g., our representation of the concept “car” consists of our previous experiences interacting with cars; Barsalou, 1999), it seems likely that some kinds of interactions are more influential than others. It is not yet known whether sensorimotor experience is always linked to the representation of a word and is beneficial for language learning regardless of whether this sensorimotor knowledge is directly involved in the specific word meaning. For example, holding a pencil provides sensorimotor information about its hardness, but does this improve children’s ability to later label this object, compared to simply observing a pencil or being told the function of a pencil? It may be that any sensorimotor information leads to acquisition of a richer semantic representation, and therefore word learning is facilitated (Barsalou, 1999). However, this may not necessarily be the case, as studies examining the effects of manipulatives on learning have demonstrated that physically interacting with perceptually rich stimuli when the sensorimotor information gained through physical manipulation is not directly related to the object name can hinder, rather than facilitate, learning an object name (McNeil et al., 2009). Embodied learning experiences may be more beneficial when the sensorimotor information obtained relates to the information learned. When this is not the case, the embodied experiences may in fact alter what is required to complete the task, and thus facilitatory embodied effects are not observed. For example, in the Tare et al. (2010) study described above, the manipulations that were performed by the children did not provide sensorimotor experience that would help the children obtain knowledge about the animals. Attractive, attention-getting stimuli may not help children to learn the intended meaning of an abstract concept or a printed word, if the appealing element needs to be represented as a symbol for something else (Uttal et al., 2009).

Object labels (nouns) are typically the first part of speech that children learn (Waxman et al., 2013), and objects tend to be perceptually rich, with numerous affordances. As such, there may be circumstances where there is no incremental benefit to providing children with additional sensorimotor experience when teaching object labels. Evidence indicates that certain embodied instructional methods may only be beneficial for certain types of information. For example, de Nooijer et al. (2013) found children’s knowledge for verbs was improved when they imitated a model by gesturing during encoding or during later retrieval; however, this gesture method was only beneficial for verbs that involved some sort of object manipulation with the hands. No beneficial effect of gesturing was observed for locomotion verbs or abstract verbs. It seems likely that in order for sensorimotor experience to be beneficial for learning, this experience needs to be appropriate and relevant to the material to be learned (Kiefer and Trumpp, 2012). Of course, defining what it means for experience to be “appropriate” to word learning is something that has not yet been achieved.

A recent trend in the embodiment literature has been to emphasize the ways in which technology can be used to facilitate learning, and results suggest that the benefits of computer interaction may depend on the information to be learned. In recent research both children and adults demonstrated better letter recognition after hand writing new letters than after typing new letters (Kiefer and Trumpp, 2012). As another example, Smeets and Bus (2012) used computer storybooks to teach 4- and 5-year-old children new words. All children saw the story scenes presented, and heard the story narration, on the computer. Children either had the story read to them with certain key words repeated, had the story read to them and interacted using the mouse to find word “hotspots” in the story, or had the story read to them and at certain points they were presented with a multiple choice question about an object, with feedback. Children who responded to multiple-choice questions learned new words more accurately than those who interacted with the story to find the “hotspots” (Smeets and Bus, 2012). It seems that while there are some applications of technology that can provide embodied experience for letter and word learning, these experiences need to correspond to the information being learned.

## THE INFLUENCE OF SENSORIMOTOR EXPERIENCE ON LANGUAGE PROCESSING

Recent research has demonstrated that embodied effects can also be observed in children’s early reading comprehension. Specifically, children’s acquisition of conceptual knowledge is enhanced when they represent story information by interacting with physical objects or manipulating objects on a computer to represent story information (Glenberg et al., 2011). In one study, 6- and 7-year-old children with low reading skills read stories about a series of events (e.g., on a farm, at the zoo; Marley et al., 2010). Children were assigned to one of three conditions: children in one condition read story sentences and at certain points used toys to act out the story action from the previous sentence. Children in the second condition read story sentences and then watched the experimenter manipulate the toys to correspond with the sentences. Finally, children in the third condition simply reread each sentence a second time. Children in the first condition, who actively manipulated the toys themselves, and children in the second condition, who observed the experimenter manipulate the toys, had more accurate recall for story events in a subsequent comprehension task than did children in the third condition.

This embodied approach to reading development was later termed “moved by reading” (Glenberg, 2011), and was extended in a further study to examine the influence of interacting with technology on reading comprehension. Glenberg et al. (2011) showed that the facilitatory effect of interaction was observed even when children manipulated story objects on a computer screen by clicking and dragging with a mouse. In some instances, computer manipulation was actually more beneficial than physical manipulation. This may be because understanding the components of the story does not require information gained from direct manipulation of physical objects; that is, haptic information such as weight or information on how to manipulate specific objects was not required in order to comprehend the stories.

These findings indicate that embodied experiences with real objects manipulated by either the self or others, as well as object manipulations on a computer, can facilitate children’s language comprehension by helping them to situate the concepts from the story in experience. These manipulation activities ground the semantic and syntactic information in the sentence in action and experience, either with the physical objects, the computer objects, or through imagining. The aim of this reading program is to make reading comprehension fast and automatic, by linking written words to sensorimotor experience (Glenberg et al., 2013).

The reading studies described above examined the influence of sensorimotor experience during language comprehension; this kind of direct effect of sensorimotor interaction is often referred to as an online effect. In contrast, offline effects occur in the absence of direct interactions, and in this vein research has also demonstrated that *previous* sensorimotor experience can influence children’s language processing. For instance, developmental studies have explored offline effects of sensorimotor experience on children’s language processing during passive listening (James and Maouene, 2009), word naming (Wellsby and Pexman, in press) and sentence/picture verification tasks (Engelen et al., 2011).

James and Maouene (2009) presented 4- and 5-year-old children with auditory lists of verbs and adjectives while they were in the MRI scanner. The results indicated that areas of the brain associated with motor processing were activated when the children listened to verbs, but not when the children listened to adjectives. These results suggest that in the developing brain there is a link between sensorimotor experience and language processing, as words that are associated with action elicit activation in the corresponding motor areas of the brain.

Wellsby and Pexman (in press) examined the influence of previous sensorimotor experience on language processing in slightly older children, using a word naming task. They assessed prior sensorimotor experience using the body–object interaction (BOI) variable. BOI captures how easily a human body can interact with a word’s referent (Siakaluk et al., 2008). This variable indexes previous sensorimotor experience, and in adult word recognition studies responses tend to be faster and more accurate to words that are high in BOI (e.g., belt) than words that are low in BOI (e.g., ship); this is termed the BOI effect (e.g., Siakaluk et al., 2008; Tillotson et al., 2008). In the Wellsby and Pexman study, 6- to 10-year-old children completed a word naming task in which high and low BOI words were presented one at a time on a computer screen and children were instructed to read the words out loud. The BOI effect in children’s naming behavior was assessed using a composite measure obtained from children’s response latency and accuracy data. Results showed that younger children (aged 6- to 8-years) did not show a BOI effect, but older children (aged 8- to 10-years) showed a facilitatory BOI effect for word naming. Wellsby and Pexman proposed that for the older children, the high BOI words activated richer semantic representations based on previous sensorimotor experiences (either personal experience or observed experience with the words’ referents). These richer representations, in the context of the older children’s relatively more proficient reading systems, led to a facilitatory BOI effect. Therefore, once children have developed reasonably efficient lexical systems and sufficient sensorimotor experience with words’ referents, they are able to use previous sensorimotor experience to facilitate word reading.

There is also evidence that older children (aged 7- to 13-years-old) construct sensory simulations of the objects and situations implied in sentences (Engelen et al., 2011). In this study, children either listened to sentences (Experiment 1) or read sentences (Experiment 2). Following each sentence children viewed a picture of an object that either matched or mismatched the visual orientation implied in the sentence. Children had to determine whether the object in each picture had been mentioned in the sentence. The results for both experiments indicated that children were faster to make this judgment when the picture matched the orientation implied in the sentence and were slower for mismatching pictures. Engelen et al. suggested that their results provide support for embodied theories of language comprehension, and the findings indicate that even in children’s developing language processing systems, simulations are constructed of the objects and events described in each sentence. Thus, the Engelen et al. (2011) and Wellsby and Pexman (in press) studies both demonstrate that language processing in older children is grounded in sensorimotor information, even when processing is offline, and separated from direct sensorimotor engagement.

## ISSUES TO BE ADDRESSED

As reviewed above, some progress has been made in understanding the role of sensorimotor experience in children’s conceptual and language learning. At the same time, there are numerous issues left to be resolved, and we highlight some of these issues here.

### WHAT SPECIFIC KINDS OF SENSORIMOTOR EXPERIENCE ARE MOST RELEVANT TO CHILDREN’S CONCEPTUAL AND LANGUAGE PROCESSING?

In this review we have discussed the role of sensorimotor experience in children’s language and conceptual development. However, our construal of what constitutes “sensorimotor experience” is quite broad and included a range of sensory experiences, for instance, moving objects in space (e.g., Smith, 2005), performing actions on objects (e.g., James and Swain, 2011), getting tactile information from touching objects (O’Neill et al., 2002), general motor exploration of the environment (Bornstein et al., 2013) and visual experience watching someone else manipulate objects (e.g., Marley et al., 2010). While the term sensorimotor experience is generally used to refer to that which results from some sort of action, it can also be primarily visual or proprioceptive. As such, further research should aim to more precisely determine the specific kinds of sensorimotor experience that are beneficial for children’s learning. As mentioned, the correspondence between sensorimotor experience and the concept to be learned is likely important. In addition, examining the exact nature of children’s experience in a task, and analyzing what it is they were trying to learn may help to determine the underlying mechanisms involved (see Wilson and Golonka, 2013, for extensive discussion of the need for task analysis).

As reviewed above, there are suggestions that the type of sensorimotor experience most relevant to word learning will depend on word class. It has also been argued that the extent to which embodied effects (attributed to sensorimotor experience) emerge in language processing depends on the degree to which that form of language comprehension is embedded in the environment (Zwaan, 2014). While there is now some research with adults on the context sensitivity of embodied language processing (e.g., van Dam et al., 2010; Tousignant and Pexman, 2012) this principle has not yet been tested in children. That is, we do not yet understand development of context sensitivity, and this seems a critical element in our understanding of the developmental pathway from the sensorimotor infant to the literate older child.

### ARE THERE OTHER ASPECTS OF EMBODIMENT THAT NEED TO BE CONSIDERED?

In the embodied literature, there has been a tendency to focus on effects of overt, goal-directed actions performed by the body; there has been less emphasis on passive sensations associated with having a body in the world when we are not directly interacting with objects (Borghi and Cimatti, 2010; Sidhu et al., 2014). That is, there has primarily been a focus on bodies acting in the environment, with limited examination of *sensing* bodies. This tendency has also been evident in the child literature. In order to fully understand development of EC, future research needs to examine the mechanisms involved in children developing a sense of their body, grounded in sensation, action, and language (Borghi and Cimatti, 2010).

A related issue is the manner in which we tend to characterize language itself. According to Borghi and Cimatti (2010) language can be conceived of as a tool that allows us to interact with our environment and, by using language, we can develop a sense of our body removed from direct actions with objects. Rather than focusing simply on how words are represented in the brain as a result of sensorimotor experiences, future research needs to examine how words can be used as tools to extend our body and interact with others (Borghi et al., 2013).

### HOW DO CHILDREN LEARN ABSTRACT CONCEPTS?

The majority of the literature reviewed has focused on children’s learning about concrete concepts and language through direct sensorimotor experience interacting with objects. A major debate in the literature concerns the extent to which EC theories can explain the processing of abstract concepts (e.g., Barsalou, 2008; Mahon and Caramazza, 2008). Therefore, an examination of whether embodied experiences can help children learn abstract concepts could suggest a developmental trajectory for the acquisition of abstract concepts. One mechanism through which abstract concepts might be embodied is the semantic association to emotional states (Pulvermuller, 2013; Zdrazilova and Pexman, 2013). Abstract words can be understood and become grounded through their associated emotional and physiological experiences, which are also considered forms of embodiment (Kousta et al., 2011). Through the experience of various emotional states and situations, the meaning of abstract concepts can become grounded in embodied experience.

There is extensive research on how adults respond to emotion words in language processing tasks (e.g., Kousta et al., 2011). This work has helped clarify how the meanings of abstract words, in particular, may be grounded through emotion. To our knowledge, no parallel research has been conducted with children. Such studies could help identify, for instance, when children begin to show effects of valence in word recognition, and how this is related (or not) to understanding abstract concepts.

Of course, emotion is not the only means by which children could learn the meanings of abstract words. Borghi et al. (2011) described a training study in which adults learned the meanings of novel concrete and abstract concepts. The study tested the notion that children learn abstract meanings through verbal explanation and relationships between perceivable objects, while they learn concrete meanings through perception and action with manipulable objects. Results were consistent with these claims, and with claims about grounding of abstract meaning in language and perception (e.g., Barsalou et al., 2008; Dove, 2011). It will be important to further evaluate this proposal in future studies with children.

### THE IMPACT OF SENSORIMOTOR DEFICITS ON LANGUAGE AND CONCEPTUAL DEVELOPMENT

As mentioned, one debate in the EC literature is focused on whether sensorimotor information is essential for conceptual processing, or if it is information that is activated epiphenomenally, as a result of spreading activation. It seems likely that this debate could be constrained by additional developmental studies on the connection between children’s sensorimotor abilities and their acquisition of language and concepts. We know that advanced motor skills and exploratory behavior in infancy are related to increased academic outcomes later in life (Bornstein et al., 2013), and a link between children’s fine motor skills and vocabulary level has also been observed (Dellatolas et al., 2003), indicating that early embodied experiences have positive influences on children’s conceptual and language learning.

While the focus has tended to be on these positive associations, additional inferences could be drawn from work on the relationship between early motor skill deficits and impairments in language and conceptual processing. For instance, developmental coordination disorder (DCD) is characterized by a general impairment in motor coordination (Visser, 2003). Many children diagnosed with DCD also show problems in other sensory domains such as vision and perception, and in cognitive domains such as attention, concentration, and language. In addition, children with specific language impairment (SLI), which is characterized by atypical language development, often show fine and gross motor skill deficits (Hill, 2001). There are numerous hypotheses as to why language and motor deficits are related, including a general slowing in processing speed (Kail, 1994), a deficit in the ability to automate skills (Fawcett et al., 1996), or an abnormality in certain brain structures (Hill, 2001). A lifespan perspective of EC could help to clarify the relationship between language and motor difficulties, and by unpacking the nature of this relationship we could provide new insight on the issue of whether sensorimotor experience is necessary for conceptual and language processing.

## CONCLUSION

In order for theories of EC to fully describe conceptual and linguistic processing across the lifespan, several issues will need to be addressed, and we have outlined some of those here. Further studies need to be conducted to examine how sensorimotor processes interact with the developing linguistic and conceptual systems in order to map out the full developmental trajectory of EC. A lifespan approach to EC will involve mapping the developmental pathways (as Smith, 2013, recommends), through which sensorimotor experiences influence the acquisition of conceptual and linguistic knowledge. This kind of integration will be a challenge, but in tackling it we believe that theories of EC can be further refined.

## Conflict of Interest Statement

The authors declare that the research was conducted in the absence of any commercial or financial relationships that could be construed as a potential conflict of interest.
